# Human Mucosal Associated Invariant T Cells Detect Bacterially Infected Cells

**DOI:** 10.1371/journal.pbio.1000407

**Published:** 2010-06-29

**Authors:** Marielle C. Gold, Stefania Cerri, Susan Smyk-Pearson, Meghan E. Cansler, Todd M. Vogt, Jacob Delepine, Ervina Winata, Gwendolyn M. Swarbrick, Wei-Jen Chua, Yik Y. L. Yu, Olivier Lantz, Matthew S. Cook, Megan D. Null, David B. Jacoby, Melanie J. Harriff, Deborah A. Lewinsohn, Ted H. Hansen, David M. Lewinsohn

**Affiliations:** 1 Division of Pulmonary and Critical Care Medicine, Oregon Health & Science University, Portland, Oregon, United States of America; 2 Portland Veterans Administration Medical Center, Portland, Oregon, United States of America; 3 Department of Pediatrics, Oregon Health & Science University, Portland, Oregon, United States of America; 4 Department of Pathology and Immunology, Washington University School of Medicine, St. Louis, Missouri, United States of America; 5 Laboratoire d'Immunologie et Unité, Inserm 932, Institut Curie Paris, France; 6 Department of Molecular Microbiology and Immunology, Oregon Health & Science University, Portland, Oregon, United States of America; National Jewish Medical and Research Center/Howard Hughes Medical Institute, United States of America

## Abstract

Control of infection with *Mycobacterium tuberculosis* (Mtb) requires Th1-type immunity, of which CD8^+^ T cells play a unique role. High frequency Mtb-reactive CD8^+^ T cells are present in both Mtb-infected and uninfected humans. We show by limiting dilution analysis that nonclassically restricted CD8^+^ T cells are universally present, but predominate in Mtb-uninfected individuals. Interestingly, these Mtb-reactive cells expressed the Vα7.2 T-cell receptor (TCR), were restricted by the nonclassical MHC (HLA-Ib) molecule MR1, and were activated in a transporter associated with antigen processing and presentation (TAP) independent manner. These properties are all characteristics of mucosal associated invariant T cells (MAIT), an “innate” T-cell population of previously unknown function. These MAIT cells also detect cells infected with other bacteria. Direct ex vivo analysis demonstrates that Mtb-reactive MAIT cells are decreased in peripheral blood mononuclear cells (PBMCs) from individuals with active tuberculosis, are enriched in human lung, and respond to Mtb-infected MR1-expressing lung epithelial cells. Overall, these findings suggest a generalized role for MAIT cells in the detection of bacterially infected cells, and potentially in the control of bacterial infection.

## Abstract 2

A first indication of the biological role of mucosal associated invariant T (MAIT) cells reveals that this discrete T cell subset is broadly reactive to bacterial infection. In particular MAIT cells recognize *Mycobacterium tuberculosis*-infected lung airway epithelial cells via the most evolutionarily conserved major histocompatibility molecule.

## Author Summary

About one-third of the world's population is infected with *Mycobacterium tuberculosis* (Mtb), yet thanks to a robust immune response most infected people remain healthy. CD8 T cells are unique in detecting intracellular infections. Surprisingly, Mtb-reactive CD8 T cells are found in humans with no prior exposure to Mtb. We show that mucosal associated invariant T (MAIT) cells, which have no previously known in vivo function, make up a proportion of these Mtb-reactive CD8 T cells and detect Mtb-infected cells via a specific major histocompatibility molecule called MHC-related molecule 1, which is evolutionarily conserved among mammals. Mtb-reactive MAIT cells are enriched in lung and detect primary Mtb-infected lung epithelial cells from the airway where initial exposure to Mtb occurs. We go on to show that MAIT cells are not specific for Mtb since they can detect cells infected with a variety of other bacteria. Curiously, Mtb-reactive MAIT cells are absent in the blood of individuals with active tuberculosis. We postulate that MAIT cells are innate detectors of bacterial infection poised to play a role in control of intracellular infection.

## Introduction

*Mycobacterium tuberculosis* (Mtb), which causes tuberculosis (TB), remains a leading cause of infectious disease mortality worldwide [1]. The majority of TB cases are exclusively pulmonary, suggesting a need for mucosal immunity in the control of Mtb. Th1-type immunity, including strong CD4^+^ Th1 cell and CD8^+^ T-cell responses, mediates control of Mtb infection [2]. Though many functions of CD4^+^ Th1 cells and CD8^+^ T cells are redundant, CD8^+^ T cells contrast with CD4^+^ cells in their ability to recognize MHC class II-negative cells and preferentially recognize cells heavily infected with Mtb [3]. In humans, Mtb-specific CD8^+^ T cells are present at high frequencies in both Mtb-infected and uninfected individuals [4],[5]. The presentation of peptide antigen bound to HLA-A, B, or C to CD8^+^ T cells is well characterized [4],[6] and has been termed HLA-Ia or classical antigen presentation.

Several nonclassical MHC-Ib (HLA-Ib) systems have been described as well. In general, these systems utilize molecules of limited polymorphism to present antigens uniquely characteristic of an infectious pathogen. Examples include presentation of short formylated peptides by mouse H2-M3 [7], presentation of lipids and glycolipids by human group 1 CD1 (CD1a–c) molecules [8]–[11], and the presentation of bacterial glycolipids by CD1d [12],[13]. In some cases these nonclassically restricted T cells have been found at high frequency prior to pathogen exposure, suggesting an innate role. In our previous studies we have determined that human neonates have high frequencies of innate Mtb-reactive thymocytes that are not restricted by classical HLA-I molecules [14]. Functionally, such cells could either provide a direct role in the control of intracellular infection or could facilitate the acquisition of adaptive immunity. In humans, Mtb-reactive group 1 CD1 [15] and HLA–E restricted CD8^+^ T cells [16] have been described.

We have observed that all individuals regardless of exposure to TB have CD8^+^ T cells capable of recognizing Mtb-infected cells [4],[5],[14]. Moreover, a proportion of these CD8^+^ T cells can be defined as nonclassically restricted [5]. Therefore, to define the relative contribution of classically versus nonclassically (NC) restricted CD8^+^ T cells, we used limiting dilution analysis (LDA) to characterize human, Mtb-specific CD8^+^ T cells in those with TB, those with latent TB infection (LTBI), and those with no evidence of prior exposure to Mtb. We show that CD8^+^ T-cell clones from individuals infected with Mtb are primarily HLA-Ia restricted. In contrast, NC restricted CD8^+^ T-cell clones that are neither HLA-Ia nor CD1-restricted, predominate in Mtb-uninfected donors but are nevertheless present in all donors. Furthermore, we demonstrate that these NC restricted CD8^+^ T-cell clones are restricted by MHC-related molecule 1 (MR1), an HLA-Ib molecule that displays striking evolutionary conservation in mammals [17]. These human Mtb-reactive T cells recognize Mtb-infected dendritic cells (DCs) and lung epithelial cells. Moreover, we show that Mtb-reactive mucosal associated invariant T (MAIT) clones cross react with cells infected with other mycobacteria as well as other bacteria such as *Escherichia coli*, *Salmonella typhimurium*, and *Staphylococcus aureus*. These clones express the semi-invariant Vα7.2 T cell receptor, are activated in a manner independent of the transporter associated with antigen processing and presentation (TAP), and have a mucosal homing phenotype. These phenotypic data lead us to designate these cells as MAIT cells [18],[19], a cell type with no previously known physiological function.

Additionally, we demonstrate that infection with Mtb induces cell surface expression of MR1 on lung epithelium. Furthermore, Mtb-reactive MAIT cells are enriched in human lung and respond to Mtb-infected lung epithelial cells. Finally, we have performed direct ex vivo analysis of Mtb-reactive MR1-restricted MAIT cells, and find they are present at lower frequencies in the blood of those with active TB. These findings suggest that MAIT cells could play a direct role in the control of bacterial infection.

## Results

### Mtb-Reactive, NC Restricted CD8^+^ T Cells Predominate in TB-Uninfected Individuals

In humans, direct ex vivo analysis of Mtb-specific CD8^+^ T cells reveals a strong association of HLA-Ia–restricted responses and infection with Mtb [4],[20]. Nonetheless, NC HLA-I–restricted CD8^+^ T cells comprise a substantial proportion of the overall response to Mtb in Mtb-infected individuals [4],[5],[20]. In individuals with no evidence of infection, we have consistently found high frequency CD8^+^ T cell responses against Mtb-infected DCs. To address the hypothesis that NC restricted CD8^+^ T cells comprise the dominant response in those without Mtb infection we performed LDA [5] using CD8^+^ T cells stimulated with Mtb-infected DCs. LDA was performed on individuals with no evidence of Mtb infection (uninfected controls, *n* = 5), individuals with evidence of latent infection with Mtb (LTBI, *n* = 5), and individuals with clinical TB (active TB, *n* = 6). From each of the 16 individuals, we screened an average of 128 clones per donor (Table 1) for their ability to specifically release interferon-γ (IFN-γ) in response to a panel of Mtb-infected but not uninfected targets.

**Table 1:** Nonclassical CD8^+^ T-cell clones predominate in TB-uninfected individuals.

Donor Status	Donor ID	Frequencies of Mtb-specific CD8^+^ cells^a^	*n* Clones Screened	Percent Classical	Percent Nonclassical
Active	D431	1/403	109	60	40
	D432	1/1156	191	50	50
	D435	1/664	17	24	76
	D466	1/528	167	95	5
	D480	1/418	192	59	41
	D481	1/618	159	96	4
*n* = 6	—	—	—	64%	36%
LTBI	D426	1/6956	24	0	100
	D443	1/1002	7	43	57
	D450	1/1102	192	16	84
	D454	1/1818	192	70	30
	D504	1/1978	192	16	84
*n* = 5	—	—	—	29%	71%
Uninfected	D403	1/2148	92	16	84
	D470	1/1774	192	4	96
	D462	N.D.	86	18	83
	D427	1/7568	192	27	73
	D497	1/3126	53	10	90
*n* = 5	—	—	—	15%	85%

a These frequencies were previously reported [4].

Abbreviations: N.D., not done.

The antigen presenting cell (APC) target groups were: autologous DCs, HLA-mismatched DCs, or HLA-mismatched macrophages. HLA-Ia restricted clones were defined as those responding only to Mtb-infected HLA matched DCs. DCs were grown in X-Vivo media to ensure expression of cell surface CD1. NC-restricted clones were defined as those responding to all three Mtb-infected APC types. As macrophages do not express CD1, NC CD1-restricted T clones were defined as those responding only to infected DCs. Using this method, we have not observed CD1-restricted T-cell clones, resulting in categorization of all the non-HLA-Ia–restricted clones as NC-restricted T cells. None of the T-cell clones were stimulated by uninfected HLA mismatched targets ruling out responses due to alloreactivity.

**Figure 1:**
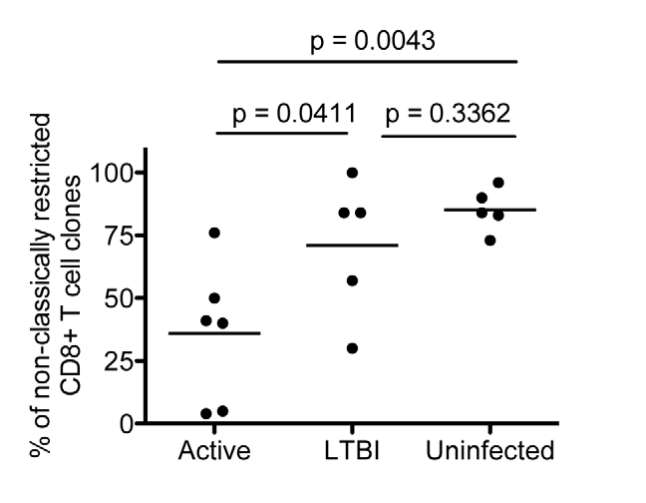
LDA of Mtb-reactive CD8^+^ T-cell clones. Scatter plot demonstrating the proportion of NC restricted CD8^+^ T-cell clones obtained from individuals in the active, LTBI, and uninfected groups. Each symbol represents the average frequency from all clones screened from an individual donor (Table 1), which was categorized as NC restricted. The nonparametric Mann Whitney one-tailed *t*-test was used to assess statistical significant differences between groups. Significant differences were detected between active and uninfected groups (*p* = 0.0043) between the active and LTBI groups (*p* = 0.0411), but not between the LTBI and uninfected groups (*p* = 0.3362).

The results from the LDA analysis are presented in Table 1 and Figure 1. The proportion of NC-restricted T-cell clones from each group of donors is presented in Figure 1. As expected, HLA-Ia–restricted CD8^+^ T-cell clones were strongly associated with TB (*p* = 0.009) (Figure 1). Nonetheless, consistent with prior observation [5], a significant proportion of CD8^+^ T-cell clones from infected individuals were NC-restricted. Furthermore, CD8^+^ T-cell clones isolated from uninfected donors predominantly displayed a NC phenotype (Figure 1).

To facilitate further analysis a representative subset of the NC-restricted T-cell clones from each donor was expanded and further characterized. Phenotypic analysis of expanded clones (*n* = 120) revealed uniform expression of CD8α and the αβ TCR (unpublished data). Additionally, we excluded potential activation by a soluble mediator by successfully using Mtb-infected paraformaldehyde-fixed DCs as stimulators. As a result, we have isolated 120 stable NC Mtb-reactive CD8^+^ αβ TCR^+^ T-cell clones.

### Mtb-Reactive NC CD8^+^ T-Cell Clones Are Restricted by the HLA-Ib Molecule MR1

**Figure 2:**
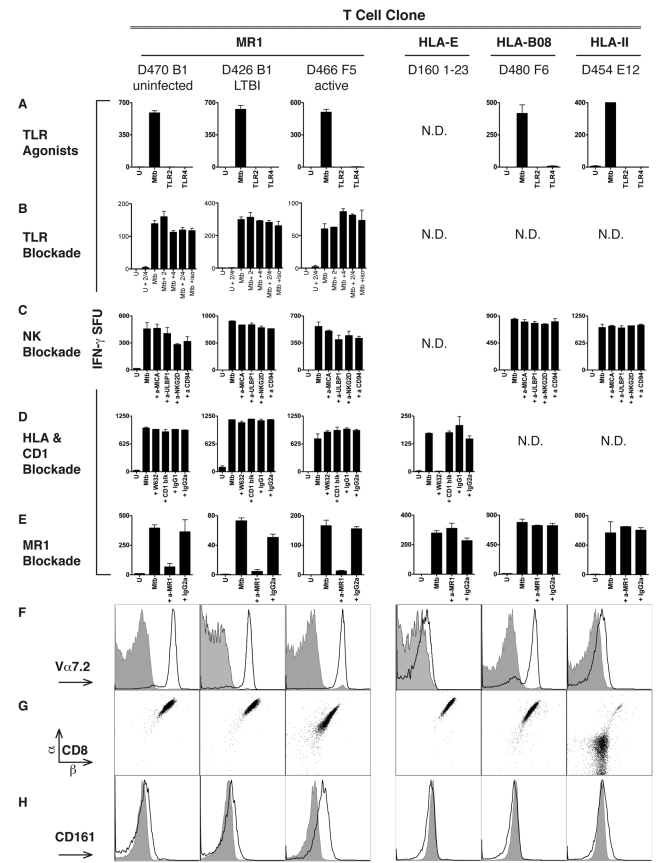
Mtb-specific NC CD8^+^ T cells are restricted by MR1. (A–E) Results of ELISPOT assays shown as IFN-γ spot forming units (SFU)/10,000 T cells in response to DCs (25,000/well) treated as described. (A) TLR agonist stimulation of DCs does not stimulate Mtb-reactive NC-restricted clones. DCs were treated (24 h) with TLR agonists specific for TLR2 (lipoteichoic acid, 10 µg/ml) and TLR4 (LPS; 100 ng/ml) at concentrations known to induce activation and cytokine production by DCs [14]. (B) TLR2 (5 µg/ml) or TLR4 (10 µg/ml) blocking antibodies were added to DCs that were uninfected or infected 1 h prior to the addition of Mtb-reactive NC T-cell clones. (C) Mtb-infected DCs were incubated with blocking antibodies (5 µg/ml) to NKG2D, ULBP1, MICA, CD94 for 1 h prior to the addition of the T-cell clones. (D) The pan HLA–I (W632) and CD1a, b, c, and d blocking antibodies were added to Mtb-infected DCs prior to the addition of T cells. (E) DCs infected with Mtb overnight were incubated with anti-MR1 blocking antibody (clone 26.5) or a mouse IgG2a isotype control (both at 5 µg/ml) for 1 h prior to the addition of T cells. (F–H) Cell surface phenotypic analyses of MR1-restricted clones and control clones. For cell surface detection, cells were incubated with antibodies specific for Vα7.2 (clone 3C10) (F), or CD8α, CD8β (G), or CD161 (H), and analyzed by flow cytometry. For (F) and (H), filled histograms represent the isotype control, bold lines represent antibody-specific staining. Columns 1, 2, and 3 represent MR1-restricted clones from different TB exposure groups: D470B1 (uninfected), D426B1 (latent), D466F5 (active), respectively. Column 4 represents HLA-E restricted clone D160 1–23 [16]. Column 5 represents HLA-B08-restricted clone D480C6 specific for the Mtb antigen CFP-10_3–11_. Column 6 represents CD4^+^ HLA-II–restricted clone D454E12 specific for the Mtb antigen CFP-10. Error bars represent the mean and standard error from duplicate wells. N.D., not done.

To explain the high proportion of NC-restricted CD8^+^ T cells, we considered three hypotheses: presentation by an HLA-Ib molecule, natural killer (NK)-receptor mediated activation, and Toll-like receptor (TLR)-mediated activation. To exclude the possibility that TLR stimulation of DCs would be sufficient to activate the NC clones, we stimulated DCs with agonists to TLR2 (lipoteichoic acid) or to TLR4 (lipopolysaccharide) [14], as both TLRs have been associated with Mtb infection [21]. TLR stimulation of DCs did not result in T-cell activation (Figure 2A). To further evaluate the possibility that TLR2 and/or TLR4 stimulation was required for the recognition of Mtb-infected targets, antibody blockade was performed (Figure 2B). Neither TLR2 nor TLR4 blockade prevented Mtb-dependent T-cell activation. However, the TLR2 and TLR4 antibodies blocked 100% and 80% of interleukin-6 (IL-6) production by DCs treated with TLR2 and TLR4 agonists respectively (unpublished data).

NK cells do not utilize a TCR but instead are regulated through opposing signals triggered through inhibitory or activating receptors. Mtb is known to induce the cell surface expression of stress molecules such as ULBP1 [22] and MICA [23], which are ligands for the activating NK receptor NKG2D. Antibody blockade of NKG2D/CD94 and the ligands ULBP1 and MICA did not alter recognition of Mtb-infected DCs by any of the T-cell clones (Figure 2C).

We next tested the hypothesis that an HLA-Ib molecule was restricting the NC CD8^+^ T-cell clones. We previously isolated Mtb-specific human CD8^+^ T-cell clones restricted by the molecule HLA–E [16]. Human Mtb-specific CD8^+^ T cells restricted by the HLA-Ib molecules CD1a, CD1b, and CD1c [24] have been extensively characterized. To assess if these HLA-Ib molecules were restricting the NC T cells, we performed antibody blockade experiments. We previously showed that addition of the pan HLA-I blocking antibody W6/32 effectively blocks the HLA–E restricted clone (D160 1–23) [16]. While addition of W6/32 blocked recognition of Mtb-infected targets by the HLA–E-restricted clone, three NC-restricted Mtb-reactive CD8^+^ T-cell clones derived from different donors were unaffected (Figure 2D). In addition to blocking all HLA-Ia molecules and HLA–E, W6/32 also blocks the HLA-Ib molecule HLA–G. As expected, the addition of blocking antibodies previously shown to block responses to CD1a, CD1b, CD1c, or CD1d also had no effect on the clones (Figure 2D). We have extended these findings to all 120 NC CD8^+^ T-cell clones isolated from the 16 donors listed in Table 2. None of the 120 clones were blocked by the addition of W6/32 or CD1 blocking antibodies (unpublished data). These results suggest that neither CD1a, CD1b, CD1c, CD1d, HLA–E, HLA–G, nor HLA-Ia molecules restrict the panel of 120 Mtb-reactive CD8^+^ NC-restricted T-cell clones. These data suggest that a common Mtb-reactive CD8^+^ T subset is present in all individuals regardless of prior exposure to Mtb.

**Table 2:** Phenotypic characterization of MR1-restricted Mtb-reactive T-cell clones.

T-Cell Clone	Donor Status	Cell Surface Phenotype		
		TCR Vα	TCR Vβ	CD161
D431G9	Active	7.2	17	−
D432BA8	Active	7.2	U	+
D466 A3	Active	7.2	20	N.D.
D466F5	Active	7.2	13.5	−
D481A9	Active	7.2	2	+
D426B1	LTBI	7.2	13.5	−
D450C8	LTBI	7.2	13.2	+
D454B6-2	LTBI	7.2	2	+
D504H11	LTBI	7.2	17	N.D.
D403C6	Uninfected	7.2	U	+
D427G10	Uninfected	7.2	U	−
D462D5	Uninfected	7.2	2	−
D470B1	Uninfected	7.2	2	−
D470C1-2	Uninfected	7.2	U	−

U, untypable indicates the Vβ chain could not be determined using the flow cytometric assay.

Abbreviations: N.D., not done.

We next postulated that the HLA-Ib molecule MR1 was the restricting allele for the NC clones. MR1 is a nonpolymorphic HLA-Ib molecule genetically linked with the CD1 locus in humans [25] and is the most evolutionarily conserved HLA–I molecule among mammals [26]. MR1 is required for the selection of a subset of T cells found primarily in the gut of mammals and thus named mucosal associated invariant T (MAIT) cells. The expansion of MAIT cells is dependent on the presence of gut flora suggesting that a bacterial derived or induced ligand is required for MR1-restricted T-cell expansion and activation [19]. Nevertheless, no bacterial or endogenous MR1-restricted antigen has been identified although considerable evidence supports an antigen presentation function by MR1 [27]–[29]. Furthermore, the biological role of MR1-restricted T cells is unknown, even though several parallels suggest a Natural Killer T-(NKT) cell–like regulatory role [30],[31].

As demonstrated in Figure 2E, addition of an anti-MR1 blocking antibody (26.5) [28] prior to the addition of NC clones abolished IFN-γ production by three different Mtb-reactive NC CD8^+^ T-cell clones and an additional 11 clones listed in Table 2. The addition of a different anti-MR1 blocking antibody (8F2.F9) resulted in similar blocking (unpublished data). In contrast, CD8^+^ T-cell clones restricted by HLA–E (D160 1–23) or HLA-B08 (D480F6), or a CD4^+^ HLA-II restricted clone (D454E12) were unaffected by the addition of the anti-MR1 blocking antibody (Figure 2E).

### MR1-Restricted, Mtb-Reactive CD8^+^ T-Cell Clones are MAIT Cells

We next performed phenotypic analyses of Mtb-reactive MR1-restricted T-cell clones to determine if they shared properties of previously characterized MR1-restricted MAIT cells. We selected a subset of clones representative of TB exposed (active, *n* = 5; LTBI, *n* = 4) and uninfected donors (*n* = 5) (Table 2). One defining feature of both mouse and human MR1-restricted MAIT cells is the expression of a semi-invariant TCR Vα chain: Vα7.2/Jα33 for humans and the highly homologous Vα19/Jα33 for mice, respectively. Using an antibody that labels all T cells containing the Vα7.2 chain including those that pair with the Jα33 region [32], the Vα7.2 chain was detected by flow cytometry on all 14 MR1-restricted Mtb-specific T-cell clones as well as on an HLA-B08–restricted clone, but not on the HLA–E, or HLA-II–restricted clones (Figure 2F; Table 2). Given that 14 of 14 randomly selected clones were restricted by MR1, binomial analysis suggests a high prevalence of MR1 restriction among our panel of clones (>95%). Furthermore, we performed an analysis of Vα7.2 TCR expression on an additional 28 NC clones. Here all 28 clones expressed the Vα7.2 TCR suggesting that the remaining clones are MR1-restricted. The TCR of Vα7.2-expressing MAIT cells from the gut has been associated with the expression of the Vβ2 or Vβ13 TCR β chains [31]. However, we found at least 10% of the Mtb-specific MR1-restricted T-cell clones did not express either Vβ2 or Vβ13 (Table 2 and unpublished data). To determine if Vα7.2^+^ Mtb-reactive MR1-restricted T-cell clones expressed the canonical Vα7.2/Jα33 CDR3 region, the TCR alpha encoding cDNA was cloned from six representative Mtb-reactive clones chosen on the basis of their distinct patterns of Vβ TCR usage. All six T-cell clones were found to express the hAV72 segment as expected, and five of six expressed the hAJ33 segment (Table 3). Further, all six Mtb-reactive TCRs were found to have VJ junctional heterogeneity with two N additions, as previously reported for Vα7.2/Jα33 TCRs [33],[34]. Importantly all six TCRs from Mtb-reactive T-cell clones were found to encode CDR3α loops of the same length, which is highly conserved among all mammalian Vα7.2/Jα33^+^ cells studied thus far. And finally, each of the sequences of the CDR3α loops of the five Vα7.2/Jα33^+^ Mtb-reactive T cells matched a sequence from a previously reported Vα7.2/Jα33^+^ cell of undefined restriction and antigen specificity (Table 3) [33],[34].

**Table 3:** Genotypic TCR analysis of MR1-restricted Mtb-reactive T-cell clones.

T-Cell Clone (Vβ)	CDR3	J Chain
D466 A3 (Vβ20)	CAVLDSNYQLIWGAG	hAJ33
D466F5 (Vβ13.5)	CAVRDSNYQLIQWGAG	hAJ33
D426B1 (Vβ13.5)	CAVRDSNYQLIQWGAG	hAJ33
D450C8 (Vβ13.2)	CARSDSNYQLIWGAG	hAJ33
D504H11 (Vβ17)	CASMDSNYQLIWGAG	hAJ33
D470B1 (Vβ2)	CAVNGDDYKLSFGAG	hAJ20

In humans, gut-derived MR1-restricted MAIT cells have been shown to express the CD8αα form of the CD8 co-receptor or lack CD4 or CD8 coreceptor expression [19],[34]. In peripheral blood, invariant TCR Vα7.2^+^ T cells were originally identified from and found to be overrepresented in the CD4^−^CD8^−^ fraction of T cells [33]. More recently, MAIT CD8αβ T cells have also been described [32],[35]. As shown in Figure 2G (and unpublished data), all of the Mtb-reactive MR1-restricted clones tested (*n* = 14; Table 2) coexpressed CD8α and CD8β chains. Human and mouse MR1 lack residues associated with CD8 interaction [25], such that the functional significance of coreceptor expression on Mtb-reactive MR1-restricted T cells remains to be determined. In a recent analysis of MAIT cells from blood, the canonical Vα7.2^+^ cells were associated with expression of the NK receptor CD161 [32]. We found that Mtb-reactive MR1-restricted T-cell clones cells varied in their CD161 expression (Figure 2H; Table 2), although all cells expressed the mucosal homing integrin α4β7, CD45RO, and lacked CD45RA as previously described for MAIT cells (unpublished data) [32].

**Figure 3:**
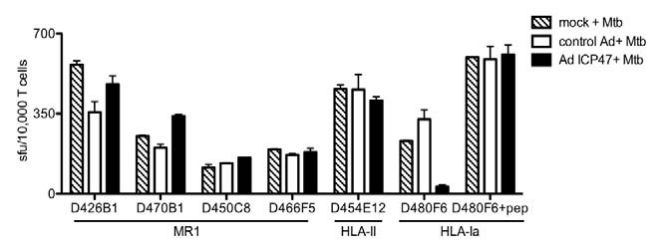
MR1-restricted recognition of Mtb-infected cells is TAP-independent. DCs autologous to D454 and expressing HLA-B08 were transduced with either a control adenoviral vector or adenoviral ICP47 using lipofectamine 2000. After 16 h, DCs were washed and either left uninfected, infected with Mtb, or pulsed with HLA-B08 specific peptide CFP10_3–11_. Following overnight incubation, T cells were added (10,000) to DCs (25,000/well) and IFN-γ production was assessed by ELISPOT. Results are representative of three independent assays. No responses were detected from T cells incubated with uninfected DCs with or without adenoviral vectors. Error bars represent the mean and standard error from duplicate wells.

Prior work with mouse and human MR1-restricted MAIT cells has demonstrated that neither HLA-II nor TAP are required for thymic selection nor for antigen processing and presentation [18],[34]. To determine if TAP transport is required for presentation to Mtb-reactive MAIT cells we used an adenoviral vector expressing the TAP inhibitor ICP47 (Figure 3) [36],[37]. When ICP47-expressing DCs were subsequently infected with Mtb, neither representative MR1-restricted clones, nor an HLA-II restricted clone were affected by TAP inhibition. In contrast, TAP blockade resulted in over 85% inhibition of the response by the CFP-10_3–11_ HLA-B08-restricted CD8^+^ T-cell clone. Hence, human Mtb-reactive MR1-restricted T cells, like previously described MAIT cells, do not require TAP for antigen processing and presentation.

### The Mtb Cell Wall is Able to Stimulate Mtb-Reactive MAIT Cells in an MR1-Dependent Fashion

To determine if an antigen from Mtb could stimulate the NC-restricted T-cell clones, we initially screened the panel of 120 stable NC clones for their ability to recognize autologous DCs loaded with the cell wall (CW) fraction from Mtb. In contrast to HLA-Ia restricted Mtb-specific T-cell clones, we found that all of the NC clones were stimulated by DCs loaded with Mtb CW (unpublished data).

**Figure 4:**
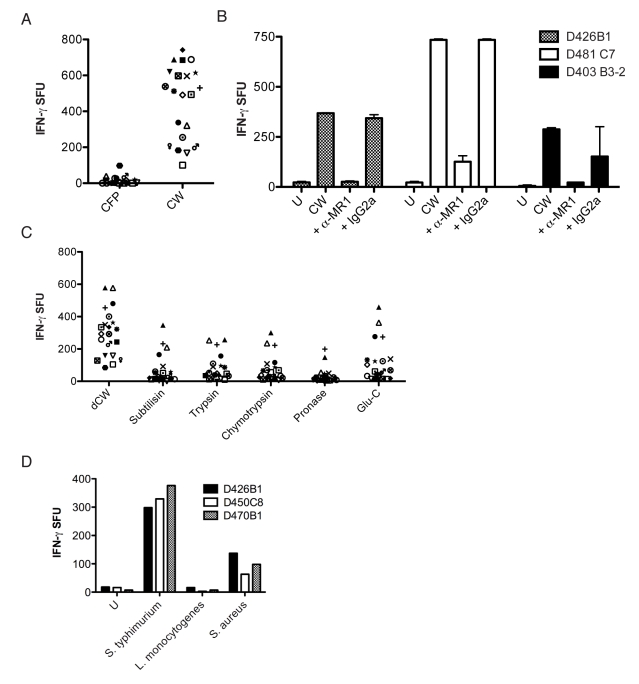
MR1 presents a protein-containing antigen from the mycobacterial cell wall. (A) CFP or CW from the Mtb strain H37Rv were added (5 µg/ml) to DCs (25,000/well) for 1 h prior to the addition of one of 21 NC clones (5,000/well) followed by IFN-γ ELISPOT assay. (B) DCs (25,000) loaded with CW overnight were incubated with anti-MR1 blocking antibody (clone 26.5) or a mouse IgG2a isotype control (both at 5 µg/ml) for 1 h prior to the addition of T-cell clones (10,000/well). (C) dCW from Mtb was treated with proteases (subtilisin, trypsin, chymotrypsin, pronase, Glu-C) and added (5 µg/ml) to DCs (25,000/well) for 1 h before the addition of 21 NC clones (5,000/well) that were tested for their ability to produce IFN-γ in an ELISPOT assay. Reversed phase- high performance liquid chromatography (RP-HPLC) chromatogram analyses were used to confirm the inactivation of proteases. No responses were detected in the absence of DCs. (D) DCs were infected with *S. typhimurium*, *L. monocytogenes*, *and S. aureus* for 1 h with a calculated moi of 145, 6, and 15, respectively. DCs were washed, antibiotics added, and DCs (25,000) were incubated with three different Mtb-reactive MAIT-cell clones (10,000/well) that were tested for their ability to produce IFN-γ in an ELISPOT assay. Results shown are similar to a minimum of three independent experiments where *S. typhimurium*, *L. monocytogenes*, *and S. aureus* were tested at a variety of moi ranging from 5 to 150.

To delineate the antigen recognized by clones we compared the ability of CW to culture filtrate protein (CFP) from Mtb strain H37Rv (courtesy of K. Dobos) to induce a response by a panel of NC-restricted CD8^+^ T-cell clones (*n* = 21 and representative of the 16 donors). As expected, the CW fraction derived from Mtb induced robust responses by all the T-cell clones (Figure 4A), whereas the CFP was not stimulatory. To determine if the presentation of Mtb CW was dependent on MR1 we performed antibody blockade. Figure 4B shows that the CW response by three distinct MR1-restricted CD8^+^ T-cell clones was dependent on MR1. To further characterize the antigen associated with the CW fraction, we subjected the CW to a variety of treatments and tested the ability of the treated fractions to induce a response by the 21 NC-restricted CD8^+^ T-cell clones (Figure 4C). We have found that delipidated CW (dCW), compared to untreated CW, is strongly antigenic. To determine whether or not the MR1 antigen was proteinaceous, dCW was subjected to proteolytic digestion with a panel of proteases. With all but three NC T-cell clones, protease treatment of the dCW abrogated the antigenic activity. The mean and standard error for the respective treatment groups were: (dCW, 298.7+/−31.72); (subtilisin, 69.38+/−20.05); (trypsin, 79.57+/−17.25); (chymotrypsin, 74+/−17.93); (pronase, 33.38+/−10.86); (Glu-C, 113.5+/−27.48). In each case the Dunn's Multiple Comparison test showed significant differences between dCW and each of the protease-treated fractions (*p*<0.05).

### Mtb-Reactive MAIT Cells Are Cross-Reactive with Other Bacteria

To determine if Mtb-reactive MAIT cells were specific for Mtb we screened known MR1-restricted clones for their ability to recognize *M. smegmatis* and *E. coli*. We found that all clones recognized DCs infected with either *M. smegmatis* or *E. coli* (unpublished data). Further evaluation of Mtb-reactive MAIT clones showed that neither adenovirus (Figure 3), nor vaccinia-infected cells (unpublished data) elicited a response by the clones. To further define the cross-reactivity of Mtb-reactive MAIT cells, three independent clones were tested for their ability to recognize DCs infected with *S. typhimurium*, *S. aureus*, and *Listeria monocytogenes*. As shown in Figure 4D, Mtb-reactive MAIT cells recognize DCs infected with *S. typhimurim* and *S. aureus*, but not *L. monocytogenes*. To confirm the lack of response by *L. monocytogenes* we infected cells at multiplicity of infection (moi) in excess of 60 and did not observe a response (unpublished data).

### Mtb-Reactive MAIT Cells Recognize Mtb-Infected Lung Epithelial Cells

**Figure 5:**
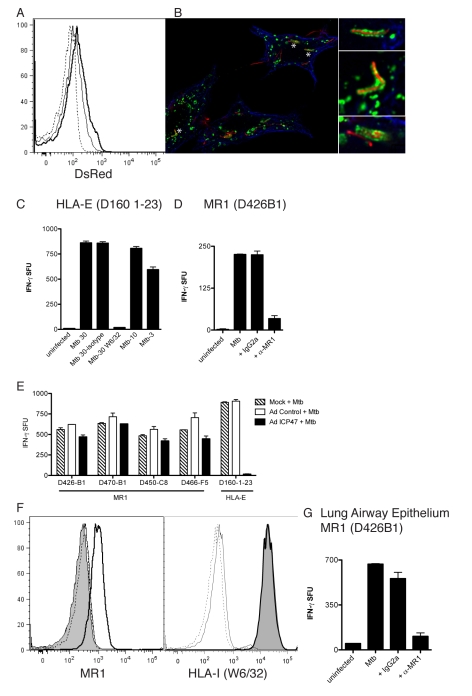
Characterization of MR1-dependent recognition of human lung epithelial cells. (A) Flow cytometric analysis of A549 cells left uninfected (dashed line) or infected with Mtb H37Rv dsRED at an moi of 10 (solid line) or an moi of 30 (bold line). (B) A549 cells were infected with Mtb (dsRED-expressing H37Rv moi of 30∶1) and incubated overnight. Cells were washed, fixed (4% paraformaldehyde), and permeabilized (0.2% saponin) before being stained for expression of Lamp1 (green) and tubulin (blue). Images were acquired on a high-resolution wide-field Core DV system (Applied Precision) with a Nikon Coolsnap ES2 HQ. One 0.5-µm Z-section is shown. Asterisks indicate magnified regions. (C) A549 cells uninfected or infected with Mtb over a range and at the moi of 30 in the presence of anti-pan HLA-I blocking antibody (W6/32) or the IgG2a isotype control (2 µg/ml each) before being used as APCs for HLA-E–restricted T-cell clone D160 1–23 in the IFN-γ ELISPOT assay. (D) A549 cells uninfected or infected with Mtb (moi 30) were used as APCs for T-cell clone D426B1 in the presence of anti-MR1 blocking antibody or the IgG2a isotype control (2 µg/ml each) in the IFN-γ ELISPOT assay. Similar results were obtained from four different MR1-restricted T-cell clones. (E) A549 cells were infected with either control vector or adenoviral ICP47. After 16 h, A549 cells were washed and left uninfected or were infected with Mtb (moi 30). Following overnight incubation, T cells were added (10,000) and IFN-γ production was assessed by ELISPOT. Results are representative of three independent assays. Error bars represent the mean and standard error from duplicate wells. (F) Flow cytometric analysis of A549 cells that were left uninfected or infected with Mtb (moi of 30). The filled histogram represents cell surface MR1 (left panel) or HLA-I (right panel) staining on uninfected cells. All lines represent cell surface staining performed on Mtb-infected A549 cells (moi 30). Dashed line, Alexa 647 secondary control; solid line, msIgG2a isotype control; bold line, cell-surface staining of MR1 (26.5)(left panel) [28], or HLA-I (W6/32) (right panel). Cell surface expression of HLA-I is identical on uninfected and infected A549 cells. (G) Human primary large airway epithelial cells were infected with Mtb (moi 30) and used as APCs (25,000/well) for MR1-restricted T-cell clone D426B1 (15,000/well) in the IFN-γ ELISPOT assay in the presence of no, anti-MR1 (26.5) or IgG2a isotype control antibodies (2 µg/ml final each). Similar results were obtained in three independent experiments.

Research on TB has traditionally focused on myeloid derived APCs such as macrophages and DCs. However, TB has the capacity to infect a variety of other cell types including epithelial cells [38]. Moreover, Mtb DNA has been detected in a variety of cell types in the lung including epithelial cells [39]. Furthermore, HLA-Ib molecules are expressed on mucosal epithelial cells [40]. Because MAIT cells are located in the gut and lung mucosa we hypothesized that MR1-restricted T cells could play a role in the detection of Mtb in lung epithelium. As shown by flow cytometry (Figure 5A) and microscopy (Figure 5B), Mtb infects the human lung epithelial cell line A549 [38]. Mtb-infection of A549 cells resulted in robust IFN-γ production by NC-restricted HLA-E (Figure 5C) and MR1-restricted T cells (Figure 5D), in a manner that was dependent on the HLA-Ib molecules HLA-E and MR1, respectively. Similarly to results shown with DCs, Mtb-infected A549 cells activated MR1-restricted, but not an HLA–E–restricted T cell [37], independently of TAP (Figure 5E).

The ability of anti-MR1 antibodies to block recognition of the Mtb-infected cells implies that T-cell activation is occurring via the cell-surface expression of MR1. Our ability to successfully block DC recognition by MR1-restricted T cells with antibody concentrations lower than 0.5 µg/ml (unpublished data) suggests that low levels of MR1 are sufficient for MAIT cell recognition. Although MR1 mRNA is ubiquitous in all human cell types [25] and MR1 protein detectable in all mouse tissues [18], cell surface expression of MR1 has not been demonstrated. Mtb infection of A549 cells resulted in the detectable cell surface expression of MR1 while HLA-I expression was unaltered by infection with Mtb (Figure 5F). We have not detected surface MR1 on DCs. We speculate that high levels of Fc receptor expression have made it difficult to discern low-level MR1 expression.

To determine whether or not primary human lung epithelial cells could act as APCs to Mtb-reactive MR1-restricted T cells, we generated human primary large airway epithelial cells from tracheal brushings [41]. As shown in Figure 5G, Mtb-infected large airway epithelial cells elicited a robust response by the MR1-restricted clone D426B1. Furthermore, this response was blocked by the addition of anti-MR1 blocking antibody (26.5) but not the isotype control (Figure 5G).

### Mtb-Reactive MR1-Dependent MAIT Cells Are Present at Reduced Frequency in the Blood of Those with Active TB

**Figure 6:**
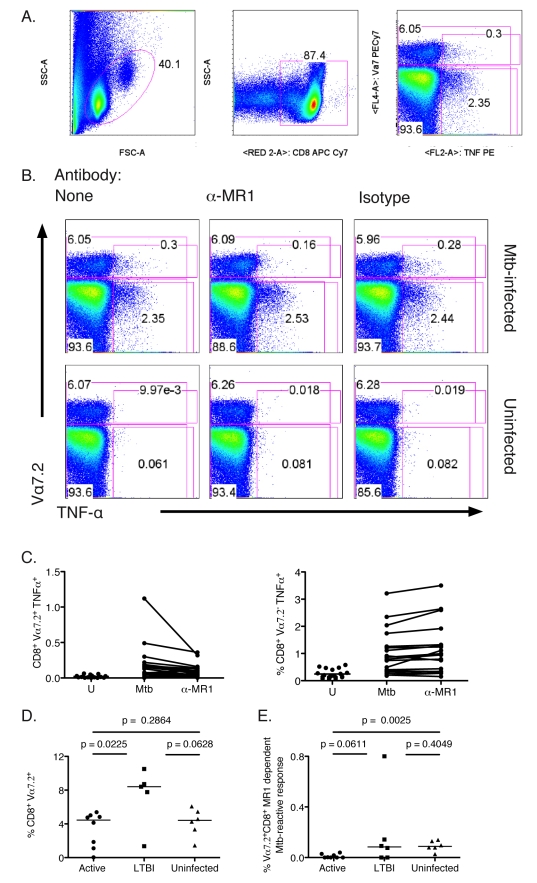
Ex vivo analysis of Mtb-reactive MAIT cells from human peripheral blood CD8^+^ T cells. T cells were isolated from PBMC using the negative pan-T cell isolation kit and then depleted of CD4^+^ T cells. The resulting T cells were incubated overnight with A549 cells that were either Mtb-infected (moi 30) or left uninfected in the presence of no, anti-MR1, or IgG2a control antibody. Golgi Stop was added for the final 6 h of the assay. Cells were surface stained for expression of the Vα7.2 TCR then fixed and permeabilized before staining for TNF-α and CD8 expression. Controls performed to test for specific TNF-α staining showed no background responses (unpublished data). (A) Gating strategy: lymphocyte gate (left), CD8 gate (middle), Vα7.2 (*y*-axis), and TNF-α expression (*x*-axis) of the CD8 gate (right). The number on the upper left of the right panel represents the frequency of CD8^+^ T cells that expressed the Vα7.2 TCR. The number in the box on the right represents the frequency of CD8^+^ T cells that produced TNF-α in response to target cells. (B) Representative FACS analysis showing the conditions tested in the assay: Mtb-uninfected or infected A549 cells in the presence of anti-MR1 antibody (26.5) or IgG2a isotype control (2.5 µg/ml each). (C) Frequency of CD8^+^ cells that produced TNF-α in response to uninfected, Mtb-infected, or MR1-blocked Mtb-infected A549 cells that either coexpressed the Vα7.2 TCR (left) or not (right). (D) Frequency of CD8^+^ T cells that expressed the Vα7.2 TCR. Statistically significant differences were observed between the active and LTBI groups. (E) Frequency of MR1-blocked CD8^+^ T cells that coexpressed Vα7.2 and TNF-α in response to Mtb-infected cells. Statistically significant differences were observed between the active and uninfected groups. Horizontal lines in (D) and (E) represent medians. The nonparametric Mann-Whitney one-tailed *t*-test was used to assess statistical significant differences between groups.

**Figure 7:**
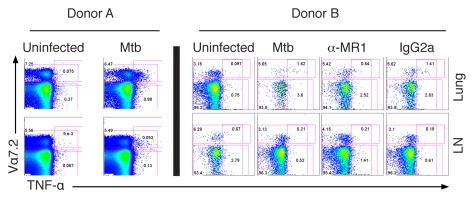
Mtb-reactive MAIT cells in the human lung. Single cell suspensions were prepared from the lung and adjacent LNs with minor modifications [52]. The intracellular cytokine staining assay was performed using magnetic-bead purified CD8^+^ T cells from the lung and LNs as described in Figure 6 legend. Only in the case of donor B were anti-MR1 or IgG2a isotype control antibodies added (2.5 µg/ml).

To determine if Mtb-reactive MR1-restricted T-cell responses are correlated with exposure to Mtb, we performed flow-cytometric ex vivo analyses of MR1-restricted, Mtb-reactive MAIT cells from subjects from all three Mtb exposure groups (uninfected, *n* = 6; LTBI, *n* = 5, active TB, *n* = 8). To enumerate these cells ex vivo, Mtb-infected A549 cells were used as APCs. A549 were chosen as APCs because they do not produce tumor necrosis factor-α (TNF-α) in response to infection with Mtb, nor elicit an allogeneic response by polyclonal CD8^+^ T cells isolated from the periphery and lung (Figures 6, 7, and unpublished data). Furthermore, in addition to IFN-γ, we have found that Mtb-reactive MAIT clones produced TNF-α in response to infected A549 cells in a manner dependent on MR1 (unpublished data).

To evaluate the nonclassical response to Mtb-infected APCs, we enriched CD3^+^ T cells from peripheral blood mononuclear cells (PBMCs) by negative selection and then depleted CD4^+^ T cells. The remaining CD8^+^ and CD4^−^CD8^−^ T cells, as the source of responding T cells, were incubated overnight with Mtb-infected A549 cells in the presence or absence of MR1-blocking antibody. Cells were surface stained to detect the Vα7.2 TCR and the CD8α coreceptor, and intracellular staining was performed to detect TNF-α production. We found that Mtb-reactive MAIT cells were uniquely CD8 single positive and CD161 negative. Therefore, subsequently, T cells were selected on the basis of forward and side scatter distribution and then selected on CD8 (Figure 6A), such that the numbers presented in Figure 6A–6E represent frequencies of total CD8^+^ cells. Figure 6B shows a representative analysis of Mtb-dependent TNF-α production from Vα7.2^+^ and Vα7.2^−^ CD8^+^ T cells. Mtb-infected A549 cells, but not uninfected A549 cells, induced TNF-α production by CD8^+^ T cells (Figure 6B and 6C). Although, Mtb-reactive responses could be detected in both the Vα7.2^+^ and negative subsets only Vα7.2^+^ CD8^+^ T cells were blocked by the addition of the anti-MR1 antibody (Figure 6B and 6C), consistent with the observation that MR1-restricted cells express the Vα7.2 TCR. On average, addition of anti-MR1 resulted in a 37% reduction in TNF-α production by Vα7.2^+^ T cells, whereas no blocking was observed from the Vα7.2^−^ T cells (*p*<0.0001). We note that in all donors a proportion of Vα7.2^−^ cells expressed the γδTCR and were activated by Mtb-infected APCs as expected from studies performed by De Libero et al. (unpublished data) [42]. Of the 19 donors, four shared at least one HLA-Ia allele with A549 cells [43]. However, three of these were from the uninfected group that were previously shown to have no detectable Mtb-specific HLA-Ia restricted T-cell responses [4].

To define the relationship of Mtb-reactive MAIT cells and host infection status, we determined the frequency of CD8^+^ T cells that expressed the Vα7.2 TCR as well as the frequency of Mtb-reactive Vα7.2^+^ T cells recognizing Mtb-infected APCs in an MR1-dependent manner (Figure 6D). The frequency of Vα7.2^+^ cells ranged from 0.039% to 10.5% of CD8^+^ T cells demonstrating considerable heterogeneity among these 19 donors. Nonetheless, a lower proportion of Vα7.2^+^ cells was present in those with active TB compared to LTBI. Figure 6E demonstrates the frequency CD8^+^ T cells that were Vα7.2^+^ MR1-dependent responses analyzed by donor infection status. When compared with uninfected subjects (mean = 0.092), those with active TB (mean = 0.011) had markedly diminished responses (*p* = 0.0025), whereas comparison with those with LTBI (mean = 0.185) revealed a less dramatic decrease (*p* = 0.0611).

### Mtb-Reactive MAIT Cells Are Enriched in the Lung

While MAIT cells have been reported in the gut lamina propria, they have also been described in the lung [19]. To test the hypothesis that MAIT cells resident in the human lung would be reactive to Mtb, the lung and adjacent lymph node (LN) were obtained from two individual organ donors whose lungs were not suitable for transplantation. Single cell suspensions from the LN and lung parenchyma were prepared and T cells were then enriched via magnetic bead positive selection. Figure 7 represents the results from intracellular cytokine staining assays performed as described in Figure 6. The frequencies of Vα7.2^+^ CD8^+^ cells in the lungs were similar to those detected in blood. However, the proportion of Vα7.2^+^ cells producing TNF-α in response to Mtb-infected APCs was notably higher (donor A, 13%; donor B, 22%) than that seen in the adjacent LN or from the blood of the 19 donors described in Figure 6 (range, 0%–10.7%; mean, 3.03; median, 2.26). These data therefore demonstrate that a substantial proportion of lung-resident MAIT cells are Mtb-reactive, and that these cells are enriched relative to the adjacent LN and peripheral blood.

## Discussion

Humans both infected and uninfected with Mtb have high frequencies of Mtb-reactive CD8^+^ T cells [4],[5]. In this study we demonstrate, by both LDA and direct ex vivo analysis that NC-restricted T cells predominate in TB-uninfected individuals. Moreover, our data suggest that MR1-restricted T cells make up a substantial proportion of the Mtb-reactive CD8^+^ NC-restricted T-cell response. Furthermore, we demonstrate that a large panel of Mtb-reactive MAIT clones are broadly reactive with mycobacterial species as well as *E. coli*.

Using LDA, we find that MR1-specific Mtb-reactive clones predominate in individuals without infection with Mtb. Our previous experience with LDA cloning has resulted in the identification of immunodominant HLA-Ia antigens and epitopes [4]. However, LDA cannot distinguish a proportionate versus absolute reduction in NC responses in individuals with active TB. Here, direct ex vivo analysis confirmed a dramatic reduction in the absolute frequency of Mtb-reactive MAIT cells in individuals with active TB.

CD8^+^ T cells restricted by both CD1 and HLA-E have been previously described but were noticeably absent from the LDA and from the resulting CD8^+^ T-cell clones. In this regard, it is possible that LDA based on positive selection of CD8^+^ T cells in conjunction with Mtb-infected DCs is not optimal for the selection of these cells. For example, CD1 molecules are downregulated by infection with Mtb [44]. Furthermore, CD1-restricted T cells may be less frequent in the CD8^+^ population [15]. Similarly, we did not isolate γδT cells [42]. As a result, the relative contribution of different HLA-Ib molecules in the host response to infection with Mtb remains to be determined.

Our findings highlight differences between MAIT cells originally characterized phenotypically, on the basis solely of TCR usage versus those characterized functionally on the basis of reactivity with Mtb. MAIT cells, as defined by the expression of the canonical Vα7.2/Jα33 TCR, appear to be universally present in humans and range in frequencies from 1% to 4% of peripheral blood T cells [32]. Here, we confirm that MR1-dependent, Mtb-reactive MAIT cells are present in the Vα7.2^+^ but not the Vα7.2^−^ population. However, Mtb-reactive MAIT cells represent a relatively small proportion of cells previously defined as MAIT cells. The observation that Mtb-reactive MAIT cells are found exclusively in the CD8 single positive CD161 negative population further defines Mtb-reactive MAIT cells as a subset of all MAIT cells. Our studies do not allow us to distinguish whether or not Mtb-reactive MAIT cells are representative of a uniform population of bacterially reactive MAIT cells or if alternate bacterial specificities exist in the Vα7.2 population.

Detailed genotypic characterization of several Mtb-reactive MR1-restricted T-cell clones reveals diversity of TCR usage. Of the six Mtb-reactive Vα7.2^+^ T cells from which the TCRα sequence was characterized, one does not express the canonical Vα7.2/Jα33 segment. Furthermore, at least 10% of the Mtb-reactive, MR1-restricted T-cell clones do not express Vβ chains previously found in preferential association with Vα7.2/Jα33 expressing PBMC of undefined restriction and antigen specificity. Whether or not differences in the TCR reflect antigenic discrimination remains to be addressed.

We speculate that environmental bacteria play a role in the selection and maintenance of human MAIT cells analogous to results previously shown in the mouse model [19]. In this regard, TCR heterogeneity could be the result of antigenic selection. We have found that DCs infected with viable mycobacteria (*M. smegmatis* and *M. bovis* bacille Calmette-Guérin [BCG]) can stimulate MR1-restricted Mtb-reactive T-cell clones (unpublished data). Environmental mycobacteria are ubiquitous and therefore may affect MAIT cell selection and maintenance. Nonetheless, we find that cells infected with nonmycobacterial microorganisms such as *E. coli*, *S. typhimurium*, and *S. aureus*, also can elicit a response by the Mtb-reactive MR1-restricted CD8^+^ T-cell clones tested thus far. At present, the molecular basis for this cross-reactivity is not known. However, recent studies using limited numbers of mouse MAIT cell hybridomas have implicated antigen presentation in MR1-restricted MAIT cell activation. This conclusion was supported by the fact that an acid eluate of purified mouse MR1 enhanced MAIT cell activation in an MR1-restricted manner [29]. Importantly, these results were obtained using uninfected cells, suggesting presentation of an endogenous antigen. Thus it is possible that MAIT cell detection of cells infected with various bacteria results from the presentation of an endogenous MR1 ligand induced by infection with various bacteria. Alternatively, it is possible that MR1 presents an exogenous antigen shared by bacteria. However, we note that cell lines overexpressing human MR1 (courtesy Ted Hansen, unpublished data) do not stimulate Mtb-reactive MAIT cells.

Based on analogy with iNKT cells, there is a precedent for recognition of either exogenous antigen or endogenous ligands by CD1d-restricted T cells [45]. However, regardless of whether an endogenous or exogenous antigen is being presented by MR1, it seems likely that MAIT cells have only a limited ability to discriminate ligands bound to MR1. Indeed the high level of activation of mouse or human MAIT cells by MR1 of different mammalian species is highly suggestive that all three components (ligand, MR1, and MAIT TCR) were highly conserved in evolution [29]. Again from analogy with CD1d-restricted presentation to iNKT cells, recent structural studies suggest they also have only limited antigen discrimination [46],[47]. The identification of physiological MR1 ligands and how they are detected by MAIT cells will clearly benefit from further studies of the extensive panel of human Mtb reactive T cells reported here.

We demonstrate here that infection with Mtb results in a modest induction of surface expression of MR1 on epithelial cells. Failure of past studies to detect surface expression of endogenous MR1 is enigmatic, since MR1 message and ER luminal MR1 protein is ubiquitously expressed in different tissues [29]. Based on these observations, it is attractive to speculate that constitutive expression of MR1 may be deleterious because of inappropriate MAIT cell activation. Such a model would be consistent with studies of induced expression of MICA/B [48]. In any case, it is also clear that very little MR1 is likely sufficient for MAIT cell activation based on our antibody blocking and cytofluorometric studies. Alternatively, it is possible that bacterial infection may alter the intracellular trafficking of MR1 and consequently determine which self or bacterial antigens are loaded and presented at the cell surface to MAIT cells. In this regard, it is important to note that in both our studies here and previous mouse studies MAIT cells are activated in a TAP-independent manner. Indeed in the mouse studies trafficking of MR1 to endosomal compartments enhanced MAIT cell activation [18]. In combination, these findings support the model that MR1 trafficking and ligand acquisition are likely altered by bacterial infection.

A surprising finding of this study has been the observation that primary human large airway epithelial cells infected with Mtb can induce a robust response by MR1-restricted MAIT cells. Following inhalation, Mtb is far more likely to encounter airway epithelium than alveolar macrophages. As a result, the capacity of lung resident MAIT cells to respond directly ex vivo to Mtb-infected lung epithelial cells suggests these cells could play a physiological role in directly controlling Mtb in the lung early in infection. Mtb-reactive MAIT cells not only produced IFN-γ but also TNF-α and granzyme (unpublished data) in response to infected targets. These effector functions could directly inhibit mycobacterial growth. Mtb-reactive MAIT cells, by IFN-γ conditioning of DCs, could also facilitate optimal priming of Mtb-specific CD8^+^ and CD4^+^ Th1 responses that are essential to control the disease in TB-exposed individuals.

Ex vivo analyses of circulating, MR1-restricted, Mtb-reactive MAIT cells demonstrate that subjects with active TB have substantially lower frequencies than those without evidence of infection with Mtb. These data suggest that Mtb-reactive MAIT cells participate in the host response to infection with Mtb. It is also possible that a similar observation would be made in bacterial pneumonia. With regard to Mtb the precise role of MAIT cells remains to be determined. We found that individuals with active TB had reduced Mtb-reactive MAIT cells. One explanation may be a genetic predisposition towards lesser expression of MR1 and/or diminished capacity to process and present bacterially derived ligands. For example, the very limited polymorphisms noted in the MR1 gene, 28 single nucleotide polymorphisms (SNPs) over a region of more than 2 MB, might allow for the delineation of SNPs associated with disease susceptibility. Alternately, it is possible that mycobacterial exposure can elicit and maintain Mtb-reactive MAIT cells. In this regard, it would be interesting to delineate the effect of BCG and/or environmental mycobacterial exposures to the prevalence of Mtb-reactive MAIT cells. Conversely, it is possible that the diminished frequencies reflect either selective migration of Mtb-reactive MAIT cells to disease sites, or their selective depletion through activation-induced cell death.

In conclusion, we have demonstrated that MAIT cells, with no previously known in vivo function, recognize bacterially infected cells. Furthermore, we demonstrate an association with Mtb exposure and/or disease status and the prevalence of Mtb-reactive MAIT cells ex vivo. Given these findings and the observation that MAIT cells are broadly reactive to bacterial infection, we postulate that MAIT cells likely play a role in the direct control of bacterial infection and/or in the subsequent acquisition of adaptive immunity to bacterial infections. By virtue of their prevalence, location, and effector functions, MAIT cells are poised to play a significant role in the control of bacterial infection.

## Materials and Methods

### Study Participants

Study participants, protocols, and consent forms were approved by the Oregon Health & Science University institutional review board. Informed consent was obtained from all participants. Uninfected individuals and individuals with LTBI were recruited from employees at Oregon Health & Science University as previously described [5]. Uninfected individuals were defined as healthy individuals with a negative tuberculin skin test and no known risk factors for infection with Mtb. Individuals with LTBI were defined as healthy persons with a positive tuberculin skin test, and no symptoms and signs of active TB. Individuals with active TB were recruited via institutional review board-approved advertisement and were self-referred from the Multnomah County TB Clinic, Portland, Oregon, US, or from the Washington County TB Clinic, Hillsboro, Oregon, US. In all active TB cases, pulmonary TB was diagnosed by the TB controller of these counties and confirmed by positive sputum culture for Mtb. Those with active TB were under the care of the local TB controller. At the time of apheresis, subjects were required to be smear and culture negative. PBMCs were isolated from whole blood obtained by venipuncture or apheresis. De-identified lung and LNs were obtained from the Pacific Northwest Transplant Bank (PNTB).

### *M. tuberculosis, Mtb-*Derived Fractions, Bacteria, and Viruses

The H37Rv strain of *M. tuberculosis* was used for all live Mtb infections (ATCC), prepared as previously described [20], and infected at moi of 30 unless stated otherwise [3]. H37RvDsRED Mtb was kindly provided by David Sherman. Fractions of the Mtb CW were obtained from K. Dobos (Mycobacteria Research Laboratories at Colorado State University, Fort Collins). Adenoviral vectors [36] were kindly provided by David Johnson (OHSU). To generate delipidated Mtb CW (dCW) 1 g of lyophilized Mtb CW was extracted at 22°C with agitation twice for 2 h with chloroform∶methanol (2∶1 v/v) (30 ml/g of CW) followed by one 18-h extraction. The 2∶1 extracted CW material was collected (27,000 *g* for 30 min) and dried under N2 and further extracted twice for 2 h followed by one 18-h extraction with chloroform∶methanol∶water (10∶10∶3 v/v/v). The resulting delipidated cell wall was dried under N2, resuspended in PBS (pH 7.4), and protein concentration determined by BCA assay (Pierce) [49]. *S. typhimurium* and *L. monocytogenes* were kindly provided by Fred Heffron and David Hinrichs, respectively. *S. aureus* was obtained from ATCC.

### Cells

A549 cells were obtained from ATCC (CCL-185). Primary large airway lung epithelial cells were derived from the trachea as previously described [41].

#### Monocyte-derived dendritic cells

Monocyte-derived DCs were prepared according to the method by Romani et al. [50]. Briefly, PBMC obtained by apheresis were resuspended in 2% human serum (HS) in RPMI and allowed to adhere to a T-75 (Costar) flask at 37°C for 1 h. After gentle rocking, nonadherent cells were removed and 10% HS in RPMI containing 10 ng/ml of IL-4 (Immunex) and 30 ng/ml of GM-CSF (Immunex) was added to the adherent cells. After 5 d, cells were harvested with cell-dissociation medium (Sigma-Aldrich) and used as APCs in assays.

### Generation and Maintenance of T-Cell Clones

Limiting dilution cloning methodology was performed as previously described with minor modifications [5]. DCs generated for use in cloning and screening T-cell clones were prepared as above with the exception that X-Vivo medium was used (BioWhittaker). Macrophages were generated using a monocyte-isolation kit (Miltenyi) and then grown for 5 d in IMDM (Invitrogen) serum-free medium. When prepared in X-Vivo medium, DCs were CD1a positive and CD14 negative, whereas macrophages grown in IMDM were CD14 positive and CD1a negative. To generate T-cell clones DCs were infected with Mtb (moi 30) overnight. CD8^+^ T cells were sorted to high purity (>99%) by FACS and were added over a range of dilutions to the infected DCs (20,000/well) in the presence of irradiated autologous feeder PBMC (1−e5/well) and rhIL-2 (10 ng/ml). T cells were screened by ELISPOT 10–14 d later. All donors from which APCs and T cells were used in these assays were genetically haplotyped (Blood System Laboratory), thereby ensuring a complete mismatch of HLA-Ia alleles when necessary for screening. T-cell clones that retained Mtb specificity were subsequently expanded in the presence of irradiated allogeneic PBMC (25×10^6^), irradiated allogeneic lymphoblastoid cell line (5×10^6^), and anti-CD3 mAb (30 ng/ml) in RPMI 1640 media with 10% HS in a T-25 upright flask in a total volume of 30 ml. The cultures were supplemented with IL-2 (0.5 ng/ml) on days 1, 4, 7, and 10 of culture. The cell cultures were washed on day 4 to remove remaining soluble anti-CD3 mAb [51] and used no earlier than day 11.

### Assays

#### IFN-γ ELISPOT assay

All IFN-γ ELISPOT assays were performed as described [16]. Estimation of the frequency of Mtb-reactive CD8^+^ T cells using the IFN-γ ELISPOT was performed as described [4].

#### Intracellular cytokine staining assay

T cells were isolated from single cell suspensions from blood, LNs, or lung using a negative selection isolation kit (Miltenyi-pan T cell kit). T cells were added to Mtb-infected or uninfected A549 cells at ratio of 3∶1 and incubated for 16 h in the presence of anti-CD28 (1 µg/ml) and CD49d (1 µg/ml). GolgiStop (BD Pharmingen) was added for the final 6 h of the assay. Cells were surface stained for expression of the Vα7.2 TCR (clone 3C10) and subsequently fixed and permeabilized with Cytofix/CytoPerm (BD Pharmingen) and stained in the presence of Perm/Wash (BD Pharmingen), with fluorochrome-conjugated antibodies to TNF-α and CD8α. Acquisition was performed with an LSRII flow cytometer with FACS Diva software (BD). All analyses were performed using FlowJo software (TreeStar).

### Reagents

Antibodies to the following molecules were used: CD1a, CD1b, CD8a CD161 TCR αβ (BD Pharmingen); CD1c (MCA694), pan HLA-I antibody (W6/32) (Serotec); anti-TNF-α (Beckman Coulter); TCR Vβ usage was determined using the IOTest Beta Mark Kit (Beckman Coulter); TCRgd (5A6.E9-Endogen); CD1d (CD1d51, kindly provided by Steven Porcelli); CD8β (GenWay); CD49d (9F10), LEAF ms IgG1, LEAF msIgG2a, Integrin B7 (FIB504) (Biolegend); MR1 (26.5) [28]; Va7.2 (3C10) [32]; CD94 (MAB1058) NKG2D (MAB139), ULBP1 (MAB1380), MICA (MAB1300) TLR2 (MAB2616), TLR (AF1478) (R&D), Lamp1 (H5G11, SCBT); Tubulin (E1332Y, Abcam); TLR agonists: Lipoteichoic Acid (Sigma); LPS (Sigma), Pam3CysK4 (InVivo Gen); Fluoromount G (Southern Biotech).

## Abbreviations

APC: antigen presenting cell
CFP: culture filtrate protein
CW: cell wall
DC: dendritic cell
dCW: delipidated CW
IFN: interferon
LDA: limiting dilution analysis
LN: lymph node
LTBI: latent tuberculosis infection
MAIT: mucosal associated invariant T cell
moi: multiplicity of infection
MR1: MHC-related molecule 1
Mtb: *Mycobacterium tuberculosis*
NC: nonclassically
NK: natural killer
PBMC: peripheral blood mononuclear cell
TAP: transporter associated with antigen processing and presentation
TB: tuberculosis
TCR: T cell receptor
TLR: Toll-like receptor
TNF-α: tumor necrosis factor-α
